# A Novel Method for High-Dimensional Anatomical Mapping of Extra-Axial Cerebrospinal Fluid: Application to the Infant Brain

**DOI:** 10.3389/fnins.2020.561556

**Published:** 2020-10-02

**Authors:** Mahmoud Mostapha, Sun Hyung Kim, Alan C. Evans, Stephen R. Dager, Annette M. Estes, Robert C. McKinstry, Kelly N. Botteron, Guido Gerig, Stephen M. Pizer, Robert T. Schultz, Heather C. Hazlett, Joseph Piven, Jessica B. Girault, Mark D. Shen, Martin A. Styner

**Affiliations:** ^1^Department of Computer Science, University of North Carolina, Chapel Hill, NC, United States; ^2^Department of Psychiatry, UNC School of Medicine, University of North Carolina, Chapel Hill, NC, United States; ^3^Montreal Neurological Institute, McGill University, Montreal, QC, Canada; ^4^Department of Radiology, University of Washington, Seattle, WA, United States; ^5^Department of Speech and Hearing Sciences, University of Washington, Seattle, WA, United States; ^6^Mallinckrodt Institute of Radiology, Washington University School of Medicine, St Louis, MO, United States; ^7^Department of Psychiatry, Washington University School of Medicine, St. Louis, MO, United States; ^8^Department of Computer Science and Engineering, New York University, New York, NY, United States; ^9^Department of Pediatrics, Center for Autism Research, Children's Hospital of Philadelphia, University of Pennsylvania, Philadelphia, PA, United States; ^10^Carolina Institute for Developmental Disabilities, UNC School of Medicine, University of North Carolina-Chapel Hill, Chapel Hill, NC, United States; ^11^UNC Neuroscience Center, University of North Carolina-Chapel Hill, Chapel Hill, NC, United States

**Keywords:** extra-axial cerebrospinal fluid, EA-CSF, Laplacian PDE, structural MRI, surface analysis, brain development, neurodevelopmental disorders, autism

## Abstract

Cerebrospinal fluid (CSF) plays an essential role in early postnatal brain development. Extra-axial CSF (EA-CSF) volume, which is characterized by CSF in the subarachnoid space surrounding the brain, is a promising marker in the early detection of young children at risk for neurodevelopmental disorders. Previous studies have focused on global EA-CSF volume across the entire dorsal extent of the brain, and not regionally-specific EA-CSF measurements, because no tools were previously available for extracting local EA-CSF measures suitable for localized cortical surface analysis. In this paper, we propose a novel framework for the localized, cortical surface-based analysis of EA-CSF. The proposed processing framework combines probabilistic brain tissue segmentation, cortical surface reconstruction, and streamline-based local EA-CSF quantification. The quantitative analysis of local EA-CSF was applied to a dataset of typically developing infants with longitudinal MRI scans from 6 to 24 months of age. There was a high degree of consistency in the spatial patterns of local EA-CSF across age using the proposed methods. Statistical analysis of local EA-CSF revealed several novel findings: several regions of the cerebral cortex showed reductions in EA-CSF from 6 to 24 months of age, and specific regions showed higher local EA-CSF in males compared to females. These age-, sex-, and anatomically-specific patterns of local EA-CSF would not have been observed if only a global EA-CSF measure were utilized. The proposed methods are integrated into a freely available, open-source, cross-platform, user-friendly software tool, allowing neuroimaging labs to quantify local extra-axial CSF in their neuroimaging studies to investigate its role in typical and atypical brain development.

## 1. Introduction

### 1.1. General Information on CSF

**Figure 1:**
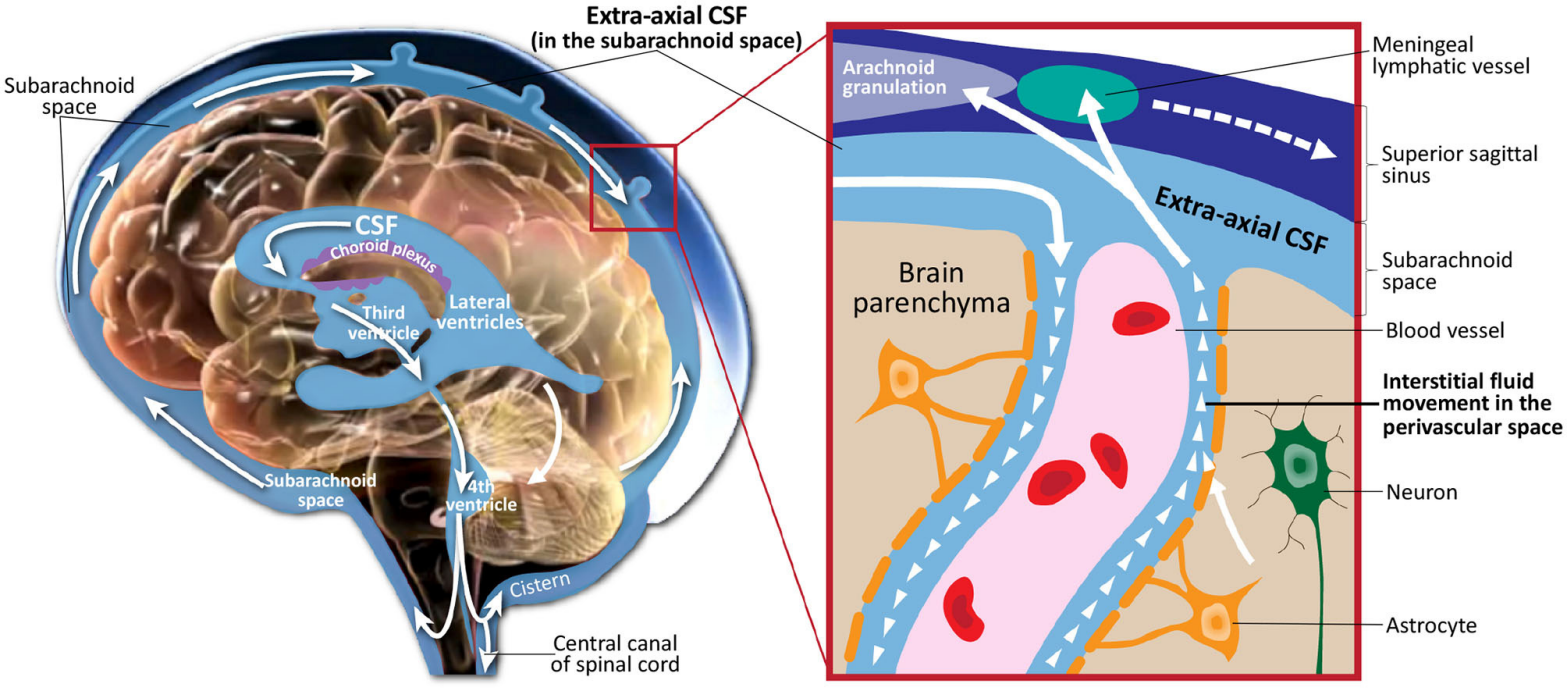
Illustration of cerebrospinal fluid circulation in the ventricles, subarachnoid space surrounding the brain, interstitial space within the brain parenchyma, and draining into the meningeal lyphatic system and arachnoid granulations. Figure adapted with permission from Shen (2018).

Cerebrospinal fluid (CSF) is a clear, colorless fluid that circulates in the brain, providing necessary mechanical and immunological protection to the brain. In addition to its protective purpose, recent findings have shown that CSF circulation plays a crucial role in brain development and function prenatally and during the lifespan (Jessen et al., 2015; Lun et al., 2015). Figure 1 illustrates CSF circulation in the subarachnoid space around the brain, spinal cord, and in the ventricles of the brain. Following CSF production by the choroid plexus in the ventricles, it circulates from the lateral, third, and fourth ventricles to the cisterns of the brain. CSF flow continues to the subarachnoid space, where it covers the cortical convexities of the brain. CSF then flows back into the parenchyma, where it interacts with the interstitial fluid within the perivascular spaces. Finally, CSF returns to the subarachnoid space, where it is absorbed through meningeal lymphatic vessels and arachnoid granulations.

It is now realized that healthy CSF circulation serves two essential functions to the brain. The first is a regulatory function by delivering growth factors and signaling molecules critical to brain development (Lun et al., 2015). Second, CSF circulation provides a filtration mechanism by removing neuroinflammatory proteins and metabolic waste byproducts of neuronal function, which would otherwise accumulate (Iliff et al., 2012). Disrupted CSF circulation has been shown to be involved in neurodegenerative conditions such as Alzheimer's disease, ischemic and traumatic brain injury, and neuroinflammatory conditions such as multiple sclerosis (Simon and Iliff, 2016). More recently, abnormalities in CSF were also linked to the onset of neurodevelopmental disorders (NDDs), including autism spectrum disorder (ASD) (Shen et al., 2013, 2017, 2018).

### 1.2. MRI-Based CSF Biomarkers

**Figure 2:**
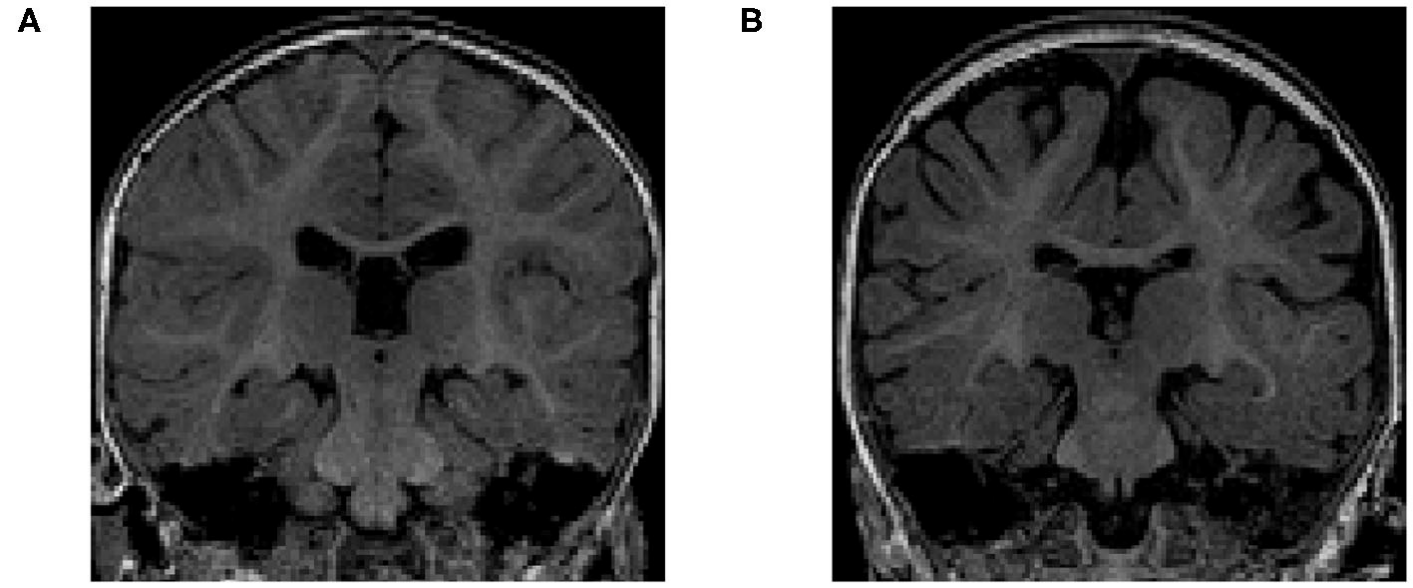
**(A)** T1-weighted MRI of a typically-developing infant with a normal MRI at 6 months of age. **(B)** T1-weighted MRI of an infant with enlarged EA-CSF volume at 6 months of age, who was diagnosed with ASD at 2 years of age. The dark regions between the brain folds and skull indicate increased volume of EA-CSF.

The volume of different CSF compartments can be accurately measured from *in vivo* structural magnetic resonance imaging (structural MRI or sMRI), which can serve as indirect markers of abnormal CSF production and absorption. Current findings in lateral ventricles (LV) volume related to ASD have indicated no consistent, significant group differences in children (Vidal et al., 2008) or adults (McAlonan et al., 2002). In contrast, there is evidence in ASD for increased volume of CSF located outside the ventricles (Hallahan et al., 2009), as well as increased volume of global CSF across the entire brain (McAlonan et al., 2004). Notably, two studies of infants at high familial risk for ASD have reported increased global volume of CSF in the subarachnoid space (Extra-Axial CSF or EA-CSF) (Shen et al., 2013, 2017). Increased EA-CSF volume at 6 months (see example in Figure 2) of age, prior to the defining behavior symptoms of ASD, was observed in infants who were later diagnosed with ASD (Shen et al., 2013). Further, EA-CSF remained abnormally elevated at 12 and 24 months of age (Shen et al., 2013). Moreover, greater EA-CSF volume at 6 months was also associated with more severe autism symptoms at the time of diagnosis at 2 years of age (Shen et al., 2013). Such EA-CSF findings were later confirmed through replication in a larger, independent cohort of infants (Shen et al., 2017). The previous EA-CSF studies relied on a novel method in infant MRIs to quantify the volume of EA-CSF in the dorsal subarachnoid space above the horizontal plane of the anterior-posterior commissure, thereby avoiding ventral regions that contain cisterns, sinuses, and vasculature that should not be classified as EA-CSF.

### 1.3. EA-CSF Quantification

The earlier results indicate that the quantification of global EA-CSF could be important in understanding the nature of brain and CSF pathology and its relation to ASD symptoms. However, the global EA-CSF measure does not provide an anatomical localization of the effect. A localized EA-CSF extraction would provide measurements suitable for localized group analysis or localized discriminative analysis. Moreover, the ability to obtain anatomically precise measures would provide greater insight into the underlying physiological and anatomical mechanisms, as well as a more fine-grained ability to detect abnormalities in NDDs. One older method to extract local measurements of EA-CSF is through voxel-based morphometry (VBM), which utilizes a statistical approach of parametric mapping (Ashburner and Friston, 2000). VBM methods are computationally efficient as they do not involve surface reconstruction of complex cortical surfaces, but the accuracy and precision of localized EA-CSF measurements from VBM are severely limited by the voxel resolution and are sensitive to volumetric registration errors, which are known to be abundantly present in most cortical areas due to the inherent cortical folding variability. Moreover, such voxel-based EA-CSF measurements cannot be easily correlated with other cortical surface-based measurements (e.g., cortical thickness and surface area) that have been shown to hold value as early biomarkers for NDDs such as ASD (Hazlett et al., 2017). Hence, the ability to extract high-dimensional surface-based local EA-CSF measurements would allow for a better understanding of how such biomarkers are related to each other, leading to optimal combinations for accurate early prediction of NDDs using deep learning techniques of multiple measures.

### 1.4. Proposed Method of Local EA-CSF

**Figure 3:**
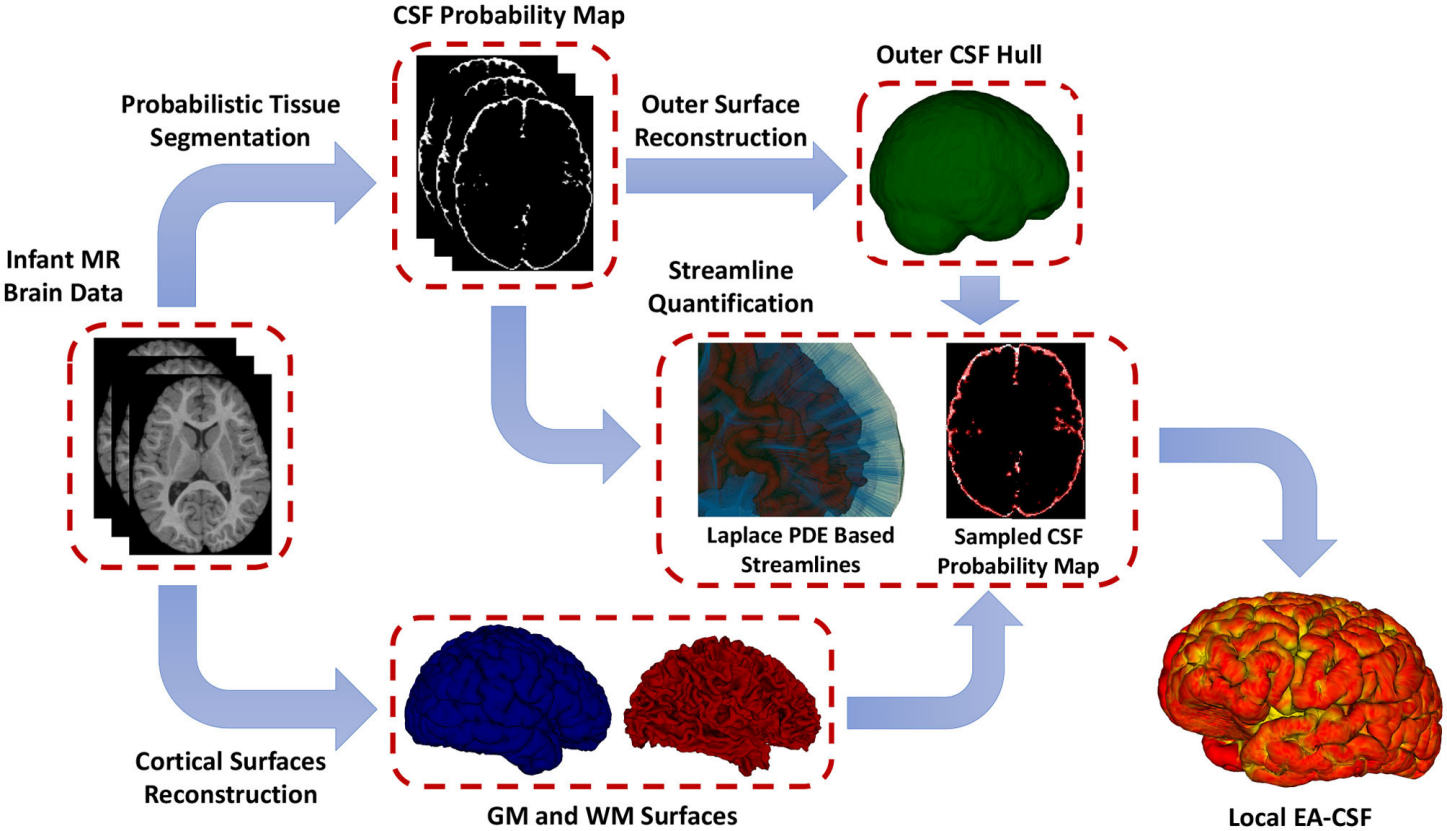
The proposed framework for the extraction of local EA-CSF from structural MRI. The proposed processing pipeline combines probabilistic brain tissue segmentation, cortical surface reconstruction, as well as streamline-based quantification to produce accurate and reliable local EA-CSF measurements.

To overcome the limitations mentioned above, as shown in Figure 3, this paper presents a novel framework for extracting surface-based local EA-CSF measurements from sMRI. The proposed framework first computes a probabilistic tissue segmentation of white matter (WM), gray matter (GM), and CSF. A hard segmentation is obtained from the tissue probability maps. These hard segmentations are then used to reconstruct polyhedral models of the outer CSF hull surface as well as the WM and GM surfaces. A Laplacian partial differential equation (PDE) is solved between a defined inner surface and the CSF hull surfaces to generate a vector field that is used to create streamlines connecting the surfaces. Along these streamlines, the CSF space is sampled, and CSF probability values are integrated to generate local EA-CSF measures at each point cortical surface. To the best of our knowledge, the proposed framework is the first to address the problem of extracting local EA-CSF measurements in a way that is suitable for localized surface-based analysis.

## 2. Methods

### 2.1. Participants and MRI Acquisition

We analyzed 153 structural MR brain images, which were obtained at 3 time points (6, 12, 24 months of age) from 51 typically-developing infants, who were at low risk for ASD (i.e., no family history of ASD, intellectual disability, or major psychiatric disorder, and who had an older sibling with typical development). These infants were scanned longitudinally at 6, 12, and 24 months of age as part of a National Institutes of Health-funded, multi-site, Autism Centers of Excellence (ACE) Network study: the Infant Brain Imaging Study (IBIS). Data collection sites had study protocols approval from their Institutional Review Boards (IRB), and all enrolled subjects had informed consent provided by parent/guardian. The MRI scans were acquired at 4 different sites (University of North Carolina at Chapel Hill, University of Washington in Seattle, Washington University in St. Louis, and Children's Hospital of Philadelphia), each equipped with 3T Siemens Tim Trio scanners (Wolff et al., 2012). The scan sessions included T1-weighted (T1w) (160 sagittal slices with TR = 2,400 ms, TE = 3.16 ms, flip angle = 8°, field of view 224 × 256) and T2-weighted (T2w) (160 sagittal slices with TR = 3,200 ms, TE = 499 ms, flip angle = 120°, field of view 256 × 256) MRI scans. All datasets had the same spatial resolution of 1 × 1 × 1 mm^3^. Only participants with scans from all three time points were included to enable an accurate longitudinal study of extracted local EA-CSF trajectories. The total sample of *N* = 51 infants (153 scans) included *N* = 32 males (96 scans) and 19 females (57 scans); see Table 1 for complete demographic information.

**Table 1:** Breakdown of ages, sex, and number of scans in the current dataset of typically developing infants from the IBIS sample.

**Number of sMRI Scans**	**All**	**Male**	**Female**
	**153**	**96**	**57**
6 Months (Mean age ± STD)	51 (6.81 ± 0.80)	32 (6.77 ± 0.81)	19 (6.89 ± 0.80)
12 Months (Mean age ± STD)	51 (12.72 ± 0.72)	32 (12.65 ± 0.66)	19 (12.84 ± 0.81)
24 Months (Mean age ± STD)	51 (24.57 ± 0.55)	32 (24.60 ± 0.58)	19 (24.51 ± 0.50)

Multiple procedures for quality control were employed to assess scanner stability and reliability across sites, times, and scanner upgrades. A Lego (Lego Group, Billund, Denmark) brick-based phantom (Fonov et al., 2011) was scanned monthly at each study site and analyzed to assess image quality and quantitatively address site-specific local distortions. Also, two adult living phantoms were scanned once per year at each scanner and after any significant scanner update. The data for these phantoms were evaluated for scanner stability across sites and time (Gouttard et al., 2008) and are also to assess stability for the local EA-CSF measure.

### 2.2. Image Processing and Surface Generation

#### 2.2.1. Initial Preprocessing

**Figure 4:**
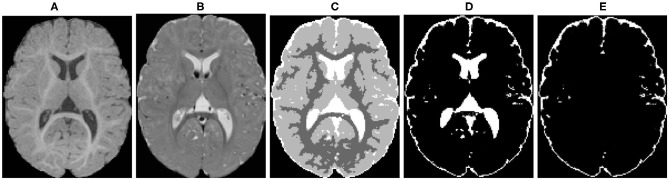
An example of the probabilistic tissue segmentation obtained for an input infant sMRI scan. **(A)** T1-weighted scan, **(B)** T2-weighted scan, **(C)** WM, GM, and CSF label map, **(D)** CSF probability map, **(E)** CSF probability map with ventricular CSF space removed.

The raw T1w and T2w brain images were corrected for intensity non-uniformity using the N4 algorithm (Tustison et al., 2010) (Figures 4A,B). Correction of geometric distortions was also applied for the optimal processing of multi-site longitudinal data (Fonov et al., 2010). T1w and T2w images were rigidly transformed to a prior pediatric 1-year-old atlas in stereotaxic space. A prior intensity growth map was applied to the 12-month T1w and T2w scans to improve the poor contrast of the WM/GM boundary from under-myelination (Kim et al., 2013).

#### 2.2.2. Skull Stripping

The brain mask necessary to perform skull stripping was performed using a multi-atlas approach that combines multiple candidate brain masks obtained via deformable registration of a prior set of atlases (each consisting of a T1w, T2w and brain mask label image). Five brain masks were utilized, namely FSL-BET (Smith, 2002), two in-house prior atlases, and two atlases of the CIVET pipeline (Kim et al., 2005). The deformable registration was computed via the ANTs registration toolkit (Avants et al., 2011) using both T1w and T2w data. The candidate brain masks were then combined, weighting each equally, to result in a majority vote fusion.

#### 2.2.3. Tissue Segmentation

Different brain tissues were then segmented using a framework of atlas-moderated expectation-maximization (EM) implemented in the AutoSeg toolkit (Wang et al., 2014). Particularly, a deformable registration was applied to propagate a prior template and prior tissue probability maps for WM, GM, and CSF from MNI space into individual T1w data (Fonov et al., 2011). Then, an EM based tissue segmentation was performed (Van Leemput et al., 1999) to obtain a label map with segmentations for WM, GM, and CSF (Figure 4C). Ventricular CSF space (lateral ventricles, third and fourth ventricles) was then removed from the CSF posterior map (Figure 4D) by deformably co-registering a single prior template with an existing ventricular area mask and using the registered mask to remove the ventricle (Figure 4E).

#### 2.2.4. Surface Reconstruction

**Figure 5:**
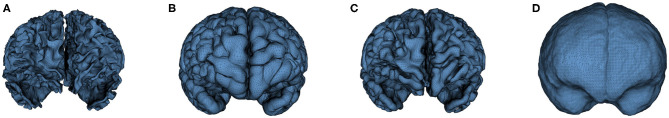
An example of the cortical surfaces reconstructed from the input infant structural MRI. **(A)** WM Surface, **(B)** GM Surface, **(C)** Middle Surface, and **(D)** CSF Hull Surface.

**Figure 6:**
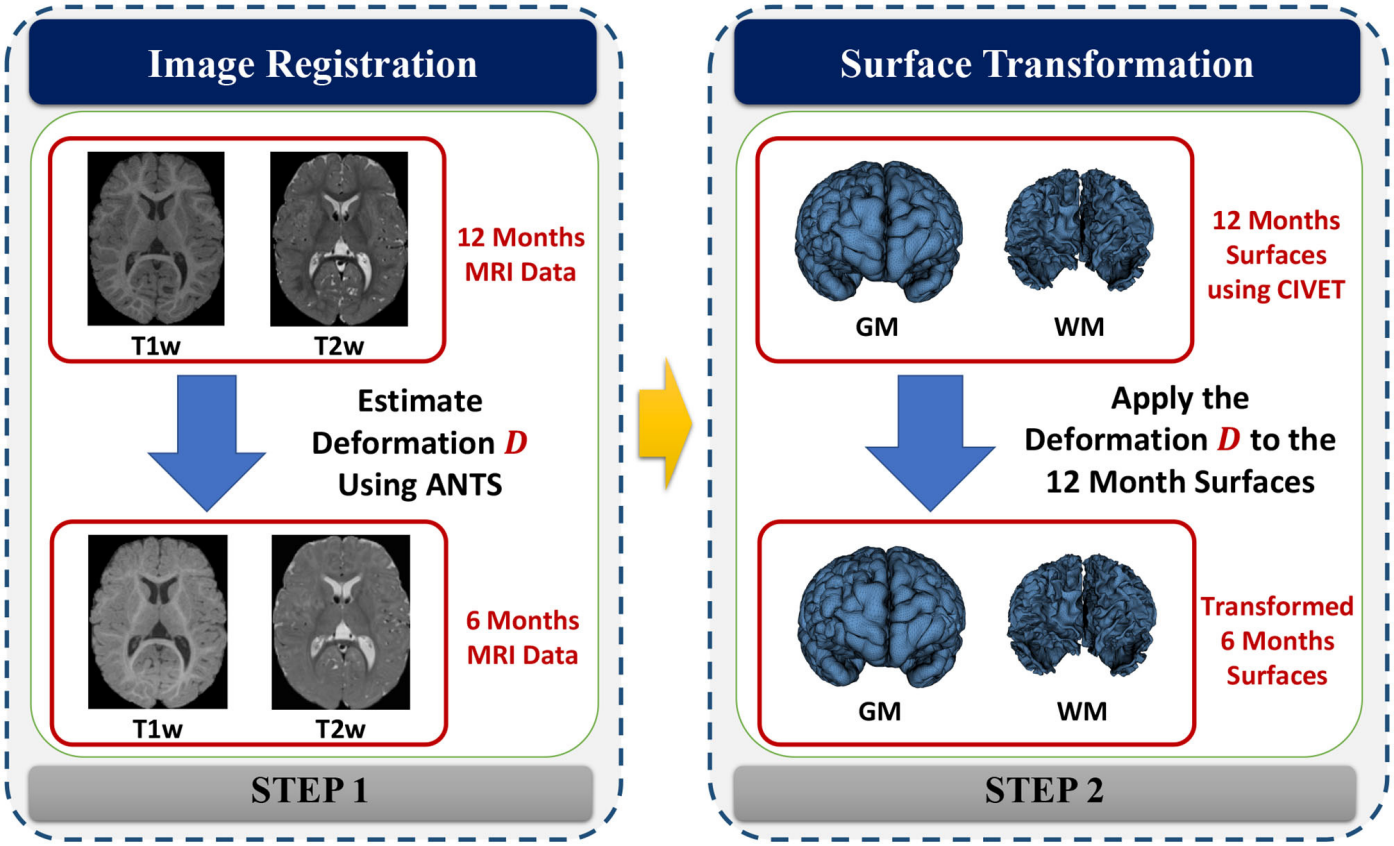
The process of generating cortical surfaces for the 6-month-old structural MRI scans.

Cortical surfaces were reconstructed with an adapted version of the CIVET workflow (Kim et al., 2005). The cortical surface model consisted of high-resolution triangle meshes (81,920 triangles and 40,962 vertices) in each hemisphere, and cortical surface correspondence among subjects was established via spherical registration to an average surface template (Robbins et al., 2003). CIVET was applied to 12 and 24 months old data following tissue segmentation with AutoSeg to construct WM and GM surfaces (Figures 5A,B). However, tissue segmentation for 6-month-old subjects did not yield reliable WM vs. GM segmentation because the WM and GM have almost the same intensity level in both T1w and T2w scans of isointense-phase infants (around 6–8 months of age). Cortical surfaces at 6 months were determined longitudinally via the corresponding 12 months visit to solve this problem. Using ANTs (Avants et al., 2011) deformable registration with normalized cross-correlation (metric radius 2 mm, Gaussian smoothing of 3 mm of the deformation map) of joint T1w and T2w data (both image sources were equally weighted), the pre-processed, brain masked MRI data of 12-month-old subjects was registered to data from the same subject at age 6 months. This registration was applied to the cortical surfaces of the 12-month-old subjects to propagate them into the 6 months space (see Figure 6). In addition to the WM and GM surfaces, a smoothed middle surface (Figure 5C) was obtained by averaging WM and GM surfaces and then two iterations of averaging based surface smoothing. Moreover, the outer CSF hull surface (Figure 5D) was generated by first dilating the intracranial mask, followed by a surface reconstruction using standard marching cubes algorithm (Lorensen and Cline, 1987) and a subsequent Laplacian surface smoothing. Finally, all the reconstructed surfaces were visually QC'ed with a surface cut overlay on the MR images by a single rater (MM).

### 2.3. Extraction of Local Extra-Axial Cerebrospinal Fluid

#### 2.3.1. Solving Laplace PDE

**Figure 7:**
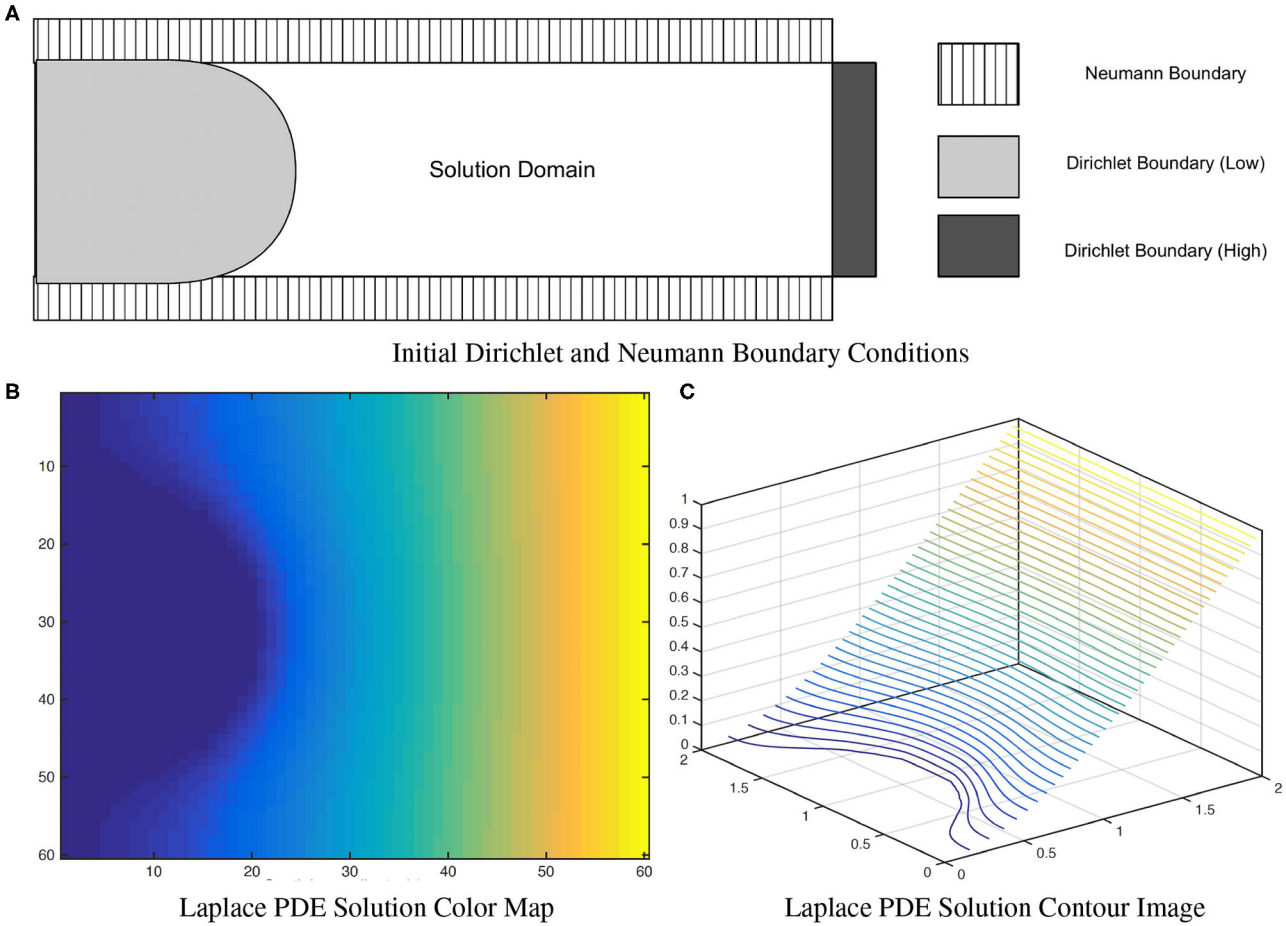
A solution of the **(A)** Laplace equation with two different boundary conditions. As observed in the color map **(B)** and the contour image **(C)**, the solution isolines are parallel to the Dirichlet boundary and perpendicular to the Neumann boundary. Figure from Lee et al. (2016).

Following the reconstruction of the cortical surfaces, the next step is to solve a Laplace's equation between the inner surface (*S*_*inner*_) and a corresponding outer surface (*S*_*outer*_). While the CSF hull surface (Figure 5D) is defined as *S*_*outer*_, in this work, we utilize the middle surface (Figure 5C) as *S*_*inner*_ instead of the GM surface to accommodate for potential surface reconstruction errors. Both *S*_*inner*_ and *S*_*outer*_ are assumed to have spherical topology, i.e., can be stretched and warped without breaking to form a sphere surface. Laplace's equation is a second-order PDE solved for a scalar field *u*(*x*) that is enclosed between boundaries *S*_*inner*_ and *S*_*outer*_. The Laplace PDE takes the following form

(1) $$\begin{array}{r} △u=\nabla^{2}u\left(x\right)=0, \end{array}$$

where *u*(*x*) = *u*_*L*_ for *x* ∈ *S*_*inner*_ and *u*(*x*) = *u*_*H*_ for *x* ∈ *S*_*outer*_. To correctly measure local EA-CSF, a boundary condition map that defines the Laplace PDE boundary condition must be defined in an anatomically consistent manner. In the solution of the Laplace PDE in the proposed framework, the solution domain is bounded by the Dirichlet condition and the Neumann condition. The Dirichlet condition specifies values of the solution itself on the boundary, while the Neumann boundary condition defines values for the first-order derivative of the solution. The interface with the Dirichlet condition defines *S*_*inner*_ and *S*_*outer*_ where streamlines start and arrive, and the Neumann condition defines an open boundary that is parallel to the streamlines (see Figure 7). A consistent boundary map generation is ensured using surface-based pre-processing steps that were applied to create a boundary label map in the image domain (Lee et al., 2016). The Laplace PDE is iteratively solved in the created image voxel grid using the Jacobi method (Causon and Mingham, 2010).

#### 2.3.2. Streamline-Based Local EA-CSF

After obtaining the Laplace PDE solution, the next step is the computation of the local EA-CSF between *S*_*inner*_ and *S*_*outer*_, which we define as CSF accumulated along the lines connecting the two surfaces. Such lines need to be orthogonal to the PDE solution isolines at each point to obtain a biologically plausible path. To achieve that, streamlines that are tangent to the normalized gradient field of the PDE solution are utilized to provide an analogy to cortical columns and to establish a one-to-one correspondence between *S*_*inner*_ and *S*_*outer*_. Such streamlines are then constructed explicitly by the integration of the Lagrangian vector field. A fourth-order Runge-Kutta (RK4) integration method (Yaakub and Evans, 1999) is used in generating the streamlines to minimize local truncation error and provide faster convergence.

**Figure 8:**
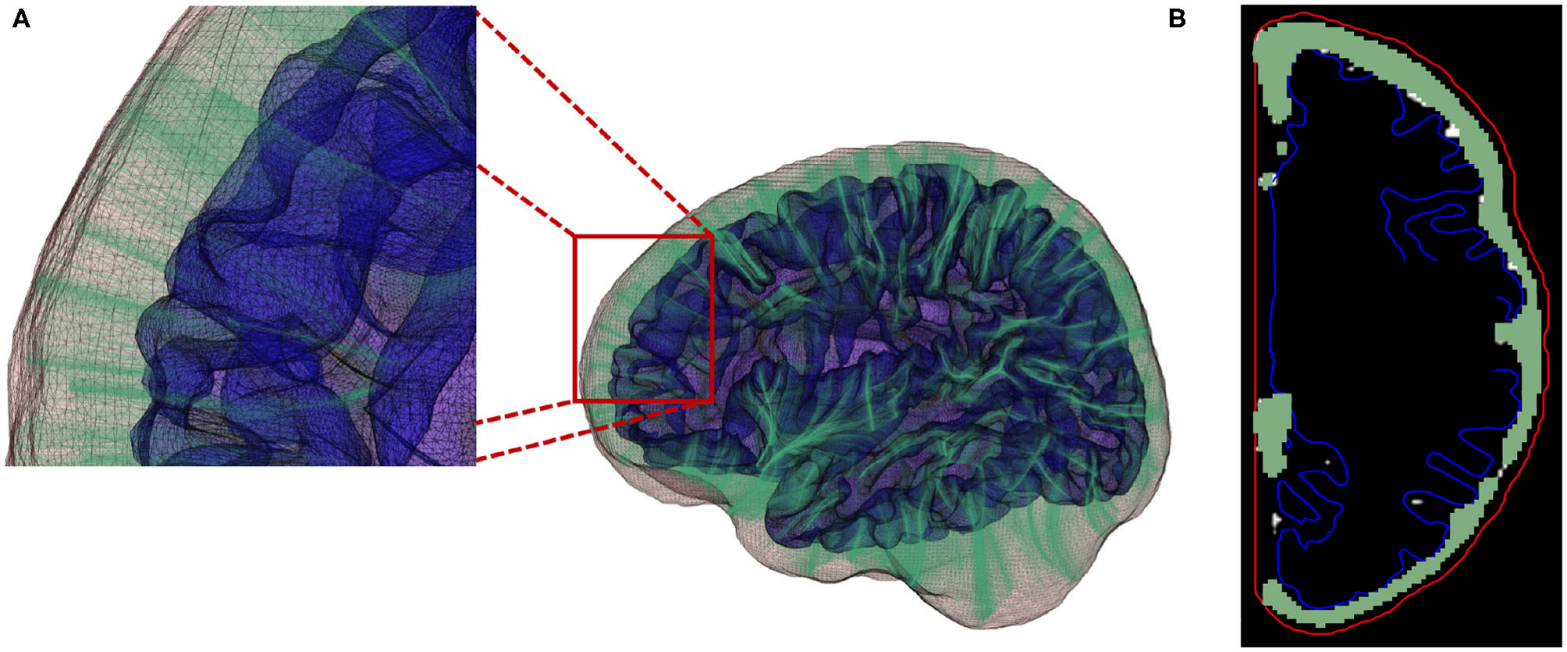
**(A)** Streamlines generated using a fourth-order Runge-Kutta (RK4) integration method. **(B)** Local EA-CSF measure is computed by accumulating CSF along the generated streamlines.

To achieve sub-voxel accuracy, we process the starting and ending segments of the streamlines to fit them perfectly within the boundary of the defined inner and outer surfaces. Finally, a local EA-CSF measure is computed for each vertex *v* by accumulating CSF probability *P* at each point *k* on the streamline *l*_*v*_ associated with *v*. A linear approximation is utilized to account for the streamlines non-uniformity

(2) $$\begin{array}{r} {\text{EA-CSF}}_{v}=\sum_{k\in l_{v}}\left(\frac{P\left(k\right)+P\left(k+1\right)}{2}\right)\times \Delta_{k}, \end{array}$$

where Δ_*k*_ representing the Euclidean distance between point *k* and the successive point *k*+1. Figure 8 provides an example for the computed streamlines and sampled CSF probability map.

As CSF values are accumulated along the streamlines, and locations/voxels with no CSF contents do not contribute to the accumulate EA-CSF measure, our proposed streamline approach is rather robust to the exact location of the inner surface as long as all CSF regions of interest lay between the inner and outer surfaces. For that purpose we chose the middle cortical surface as the inner surface (see section 2.2). Choosing the gray matter surface as inner surface would likely exclude minor EA-CSF sections due to segmentations errors of the gray matter boundary.

#### 2.3.3. Statistical Analysis

**Figure 9:**
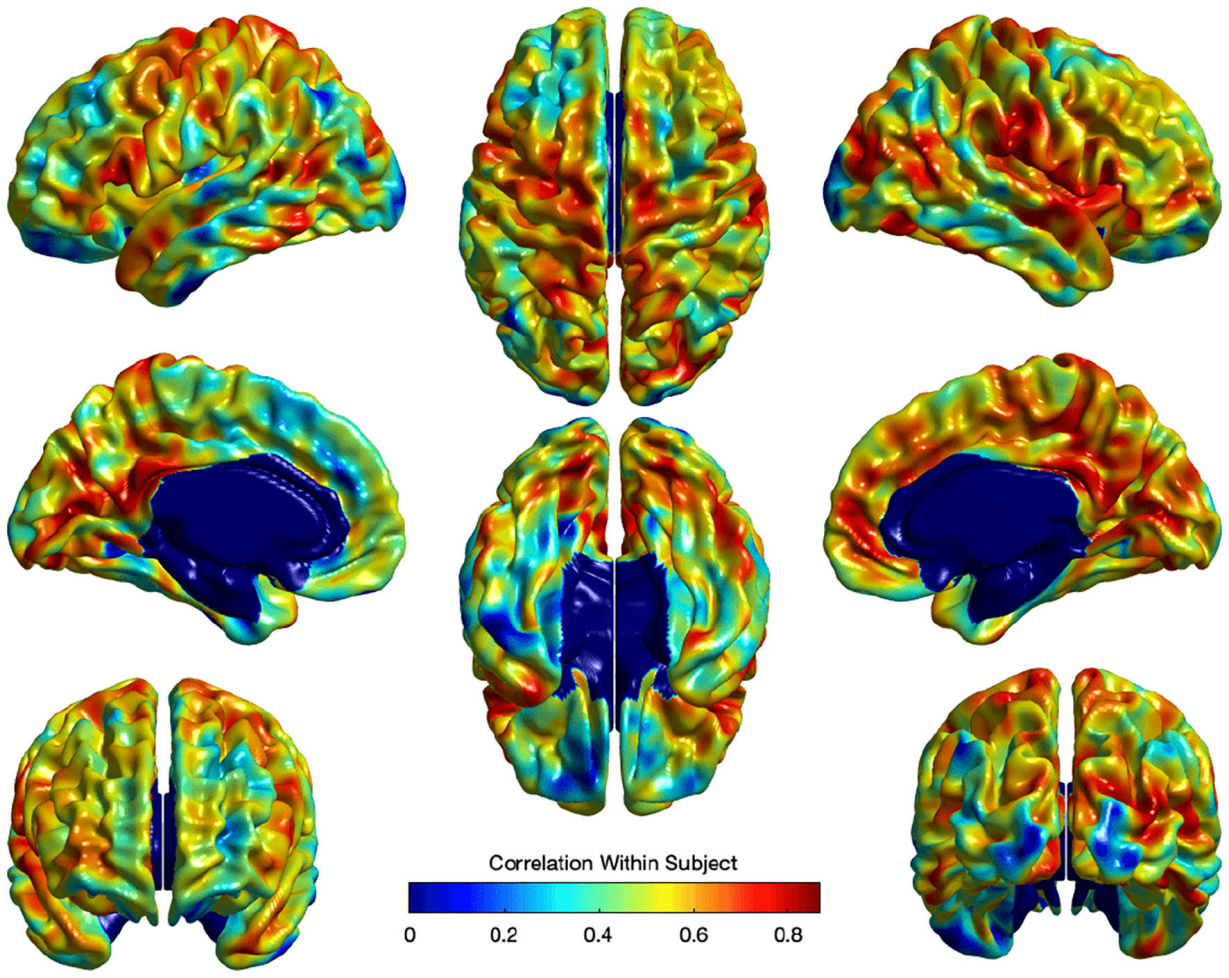
The correlation of local EA-CSF within-subject across ages 6–24 months. High correlations in the frontal, parietal, and temporal lobes were observed.

The extracted raw local EA-CSF maps were first mapped to the common MNI surface template space (Lyttelton et al., 2007) for additional processing and analysis. As an initial standard processing step in the study of cortical surface measurements, a geodesic heat kernel-based smoothing (FWHM of 20 mm) was applied to the EA-CSF maps (Chung et al., 2005). The effects of sex and age on local EA-CSF were tested using a longitudinal mixed-effects model in SurfStat, which is a toolbox for statistical analysis of cortical surface measurements applying random field theory for statistical inference (Chung et al., 2010). The longitudinal linear mixed model included a subject-specific random intercept to induce equal correlations between observations on the same subject. Slope terms were also added to model the fixed effects of sex, of age, as well as of sex and age interactions. In particular, with the local EA-CSF as the dependent variable *Y*, the following linear mixed model was fitted for each subject *i*:

(3) $$\begin{array}{r} Y_{i}=\beta_{0}+\beta_{\text{1}}{\text{Sex}}_{i}+\beta_{\text{2}}{\text{Age}}_{i}+\beta_{\text{3}}{\text{Sex}}_{i}{\text{Age}}_{i}+U_{i}+\varepsilon_{i}, \end{array}$$

where *U*_*i*_ captures estimates for the subject-specific random effect and ε_*i*_ is the independent noise term in every observation. Standard false discovery rate (FDR) (Benjamini and Hochberg, 1995) correction was applied to correct for the multiple comparisons in the model in Equation ( 3). Figure 9 illustrates the within-subject correlation of local EA-CSF across age, as revealed by the linear mixed model. High correlations are shown across most of the brain regions, particularly in the frontal, parietal, and temporal lobes. In the presence of highly correlated areas, subject-specific random effects need to be incorporated in the linear mixed model.

## 3. Experimental Results

### 3.1. Local EA-CSF Reproducibility

The stability and reliability of the proposed local EA-CSF measure were tested using a dataset with a large set of scan/rescan MRIs. Two human phantoms (young male subjects, age 26, and 27), were scanned with the same pulse sequence at four different sites using Siemens 3T Tim Trio scanners, at irregular intervals over 2.5 years. This resulted in 35 MRI scans for subject I and 31 MRI scans for subject II. The tissue segmentation, brain surface reconstruction, and computation of local EA-CSF maps were performed independently. Local EA-CSF maps were then analyzed using the local coefficient of variation (CV) as a measure of stability. The CV for a vertex *v* is defined as the ratio between the standard deviation and mean of the extracted local EA-CSF across diffident scans of the same subject:

(4) $$\begin{array}{r} CV_{v}=\frac{\sigma_{v}}{\mu_{v}}\times 100. \end{array}$$

It is noteworthy that the EA-CSF spaces in these two adult subjects were visually smaller than those we observed in our infant dataset. In general, in our experience infants have visually larger EA-CSF spaces than adults. It is thus likely our results yield a conservative estimate of the expected reproducibility in the infant settings.

**Figure 10:**
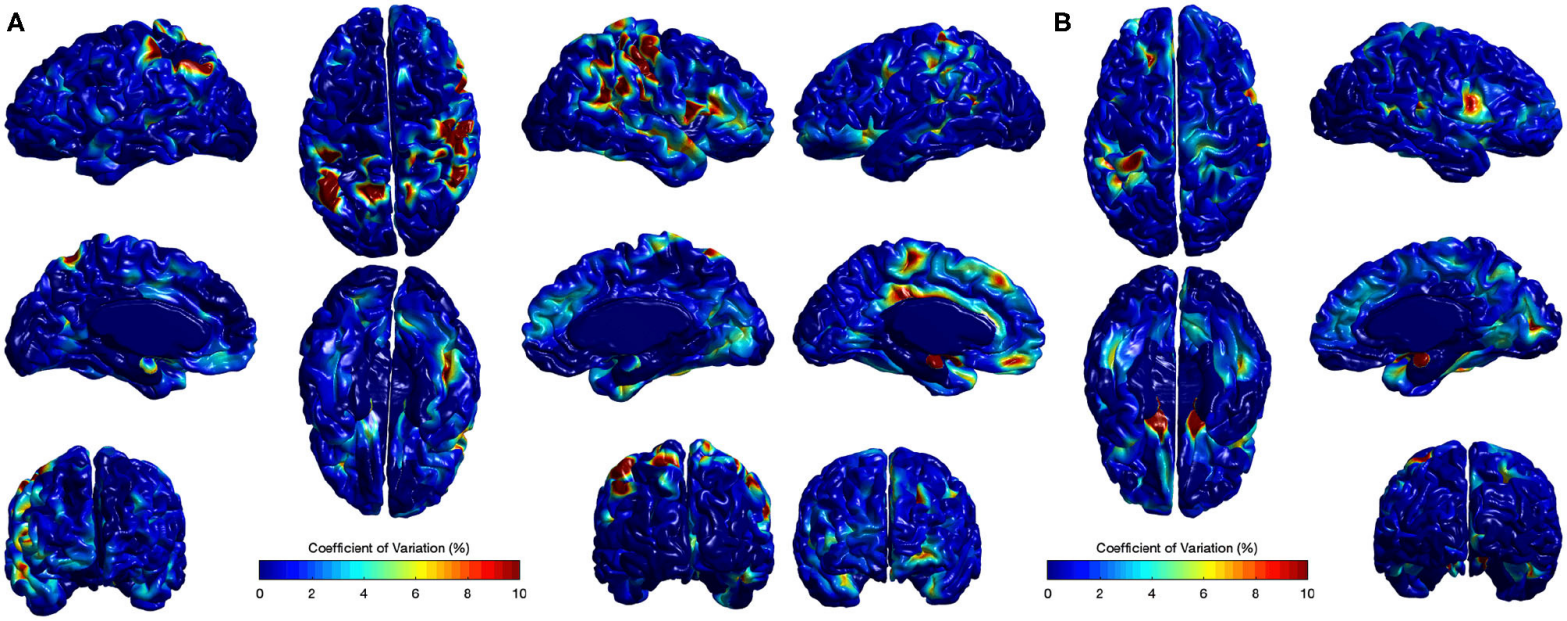
Coefficients of variation (σ_*v*_/μ_*v*_× 100) for local EA-CSF maps of two sets of adult brains. Mean coefficient of variation of 1.15 and 1.56% were observed for **(A)** Case I and **(B)** Case II, respectively. Regions with high coefficient of variation are linked to CSF segmentation issues.

The CV analysis showed excellent stability with mean across-site CV of 1.15 and 1.56% for all cortical regions in Case I and Case II respectively (Figure 10). Higher CVs were observed in few regions, including left supramarginal gyrus, left postcentral gyrus, left gyrus rectus, right postcentral gyrus, right superior temporal gyrus, and right precentral gyrus. Local EA-CSF variability in these regions was mainly linked to imperfect CSF tissue segmentation. It is worthwhile to mention that the global ICV measures showed CV values around 1% (Bryson et al., 2008; Hazlett et al., 2012) in the same human datasets. Hence, the proposed local EA-CSF extraction framework provided a stable local measure as compared to global ICV in adult brains.

### 3.2. Anatomical Mapping of Local EA-CSF Across Age

**Figure 11:**
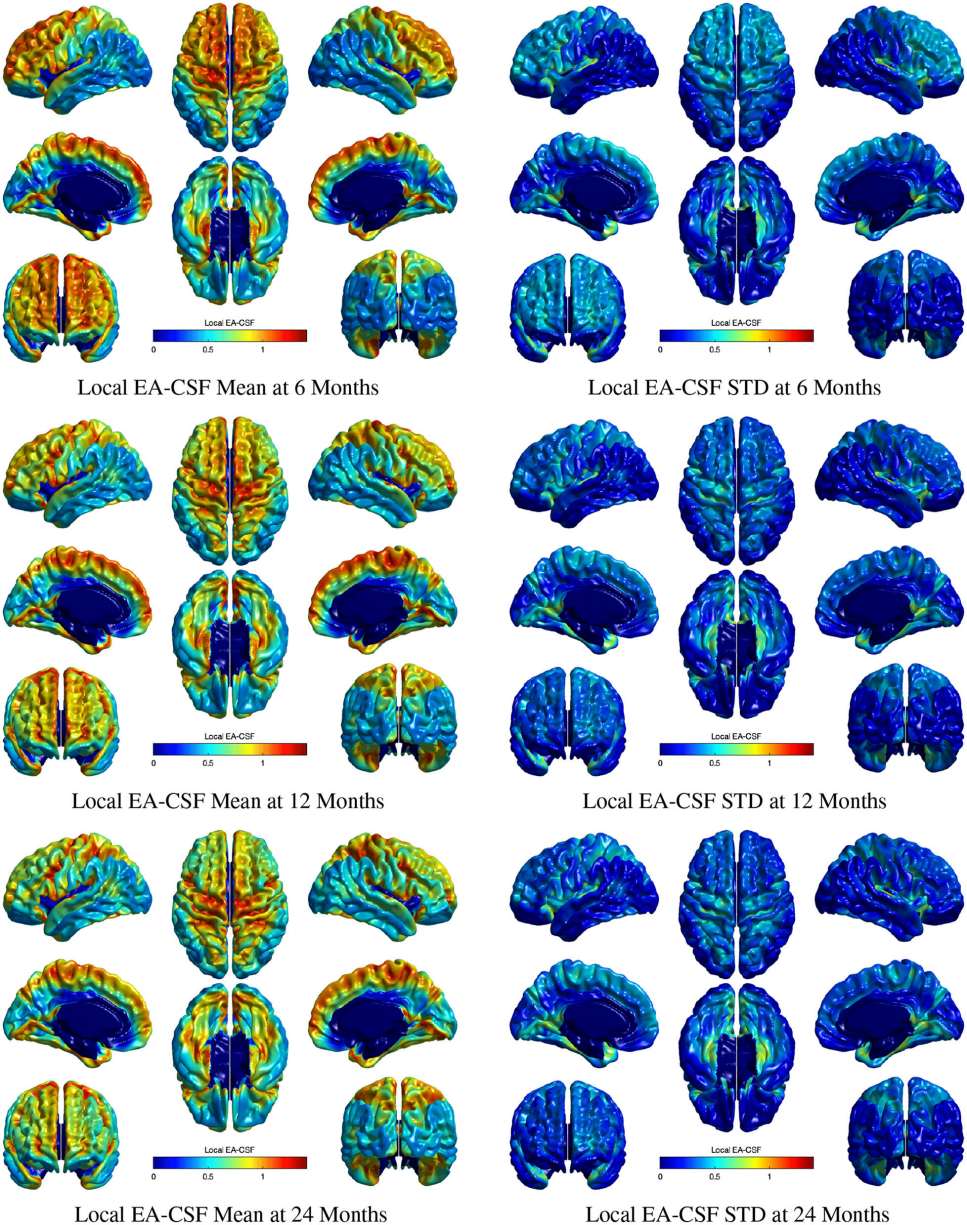
The mean and standard deviation of local EA-CSF measured at 6 months (first row), 12 months (second row), and 24 months (third row). At all ages, local EA-CSF is most abundant over the central sulcus, precentral sulcus and longitudinal fissure. The highest inter-subject variability of local EA-CSF was found in the medial and ventral temporal areas.

Figure 11 shows the mean and standard deviation of local EA-CSF maps at 6, 12, and 24 months. At all ages, local EA-CSF is most abundant over the central and precentral sulci. This observation is consistent with visual inspection of hundreds of infant MRI brain images (Shen et al., 2013, 2017). We also observed finer grained findings that are consistent at all the studied ages, such as increased EA-CSF along the superior frontal gyrus, fusiform gyrus, and the calcarine sulcus, as well as decreased EA-CSF in the inferior parietal lobule and the middle temporal gyrus. These observations are novel. As a group, this sample of typically developing infants showed anatomical consistency of the average, local EA-CSF patterns across time (left column in Figure 11). Yet, differences across individuals at each age are quite large as evident from the mean coefficients of variation of 44.6, 42.2, and 42.9% at 6, 12, and 24 months, respectively.

**Figure 12:**
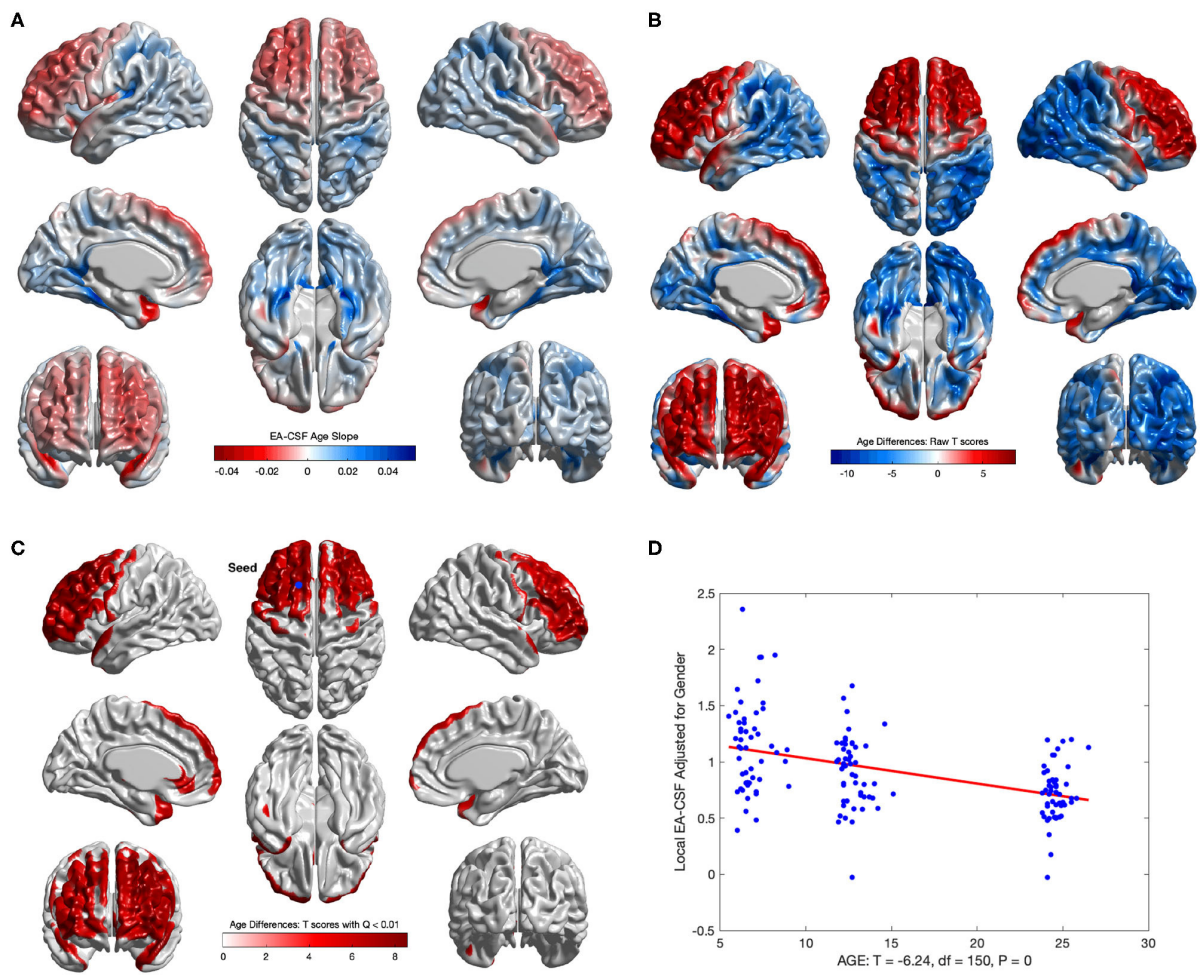
The longitudinal age effect on local EA-CSF. **(A)** Frontal regions showed a mean negative change rate of local EA-CSF of –0.011. The greatest negative change rate of –0.046 per month was observed in the left superior temporal gyrus. The corresponding **(B)** raw T-scores (red: negative age effect; blue: positive age effect) and **(C)** FDR corrected T-scores in regions with statistically significant age effect (FDR *Q* < 0.01, only regions with a negative age effect survived FDR correction); **(D)** EA-CSF plot across age at the surface location with maximum T score, (*T*_*max*_ = 8.57, located in left frontal superior gyrus).

Consistent with our previous global EA-CSF report (Shen et al., 2017), we observed a significant decrease in local EA-CSF over time. Figures 12A,D shows the linear change rates of local EA-CSF per month between 6 and 24 months of age. The mean negative change rate of all regions that showed statistically significant differences across age was –0.011 per month. Multiple regions of the frontal lobe showed significant negative change rates of EA-CSF, while the region with the greatest negative change rate (–0.046 per month) was observed in the left superior temporal gyrus. Figures 12B,C shows the FDR-corrected T scores (FDR threshold *Q* < 0.01) for the age effect resulting from the longitudinal mixed-effects model. Overall 16.9% of the brain showed a significant decrease in local EA-CSF in the first 2 years of infant's life. Local EA-CSF showed a highly negative correlation with age in most of the frontal lobe areas, including bilateral middle frontal gyrus and bilateral superior frontal gyrus. Negative correlation with age was also observed in some temporal regions, such as the bilateral superior temporal gyrus. No cortical areas showed a significantly positive association of age and the local EA-CSF.

### 3.3. Sex Differences in the Anatomical Mapping of Local EA-CSF

**Figure 13:**
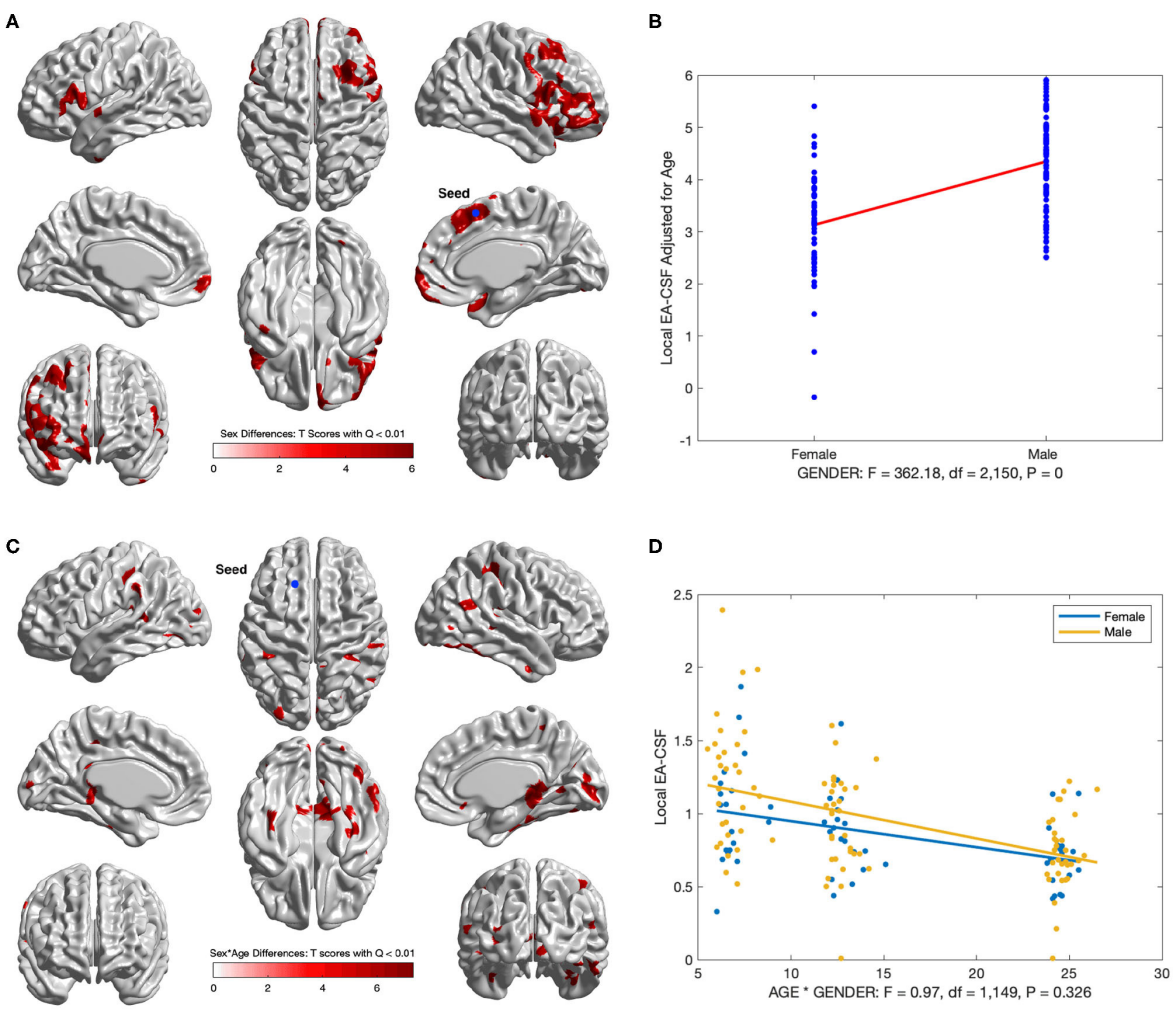
Regions with sex differences in local EA-CSF. **(A)** FDR corrected T-scores in regions with statistically significant sex effect (FDR *Q* < 0.01, red: male > female; blue: male < female). **(B)** Sex differences at location with maximum T score, (*T*_*max*_ = 6.03, located in the right superior frontal gyrus). **(C)** Regions with statistically significant sex by age interaction (FDR *Q* < 0.01). **(D)** Local EA-CSF profile at location with maximum sex by age effect (located in the left superior frontal gyrus).

Several brain regions showed a statistically significant main effect of sex across age (FDR threshold *Q* < 0.01), with higher local EA-CSF in males compared to females in regions such as: right middle frontal gyrus, inferior and medial orbital gyri, bilateral inferior frontal gyri, right insula, and right superior temporal gyrus, right supplementary motor cortex, and right superior frontal gyrus. 6.2% of the brain showed more local EA-CSF in males compared to females. There were no regions where females showed larger EA-CSF (See Figures 13A,B). It is noteworthy that our longitudinal mixed model did not covary for head size, for example, via intracranial cavity volume.

In addition to the main sex effect, brain regions showed a significant sex by age interaction, whereby local EA-CSF decreased at a faster rate in males compared to females, including: bilateral calcarine fissure, bilateral parahippocampal gyri, bilateral middle temporal gyri, right superior parietal gyrus, right precuneus. Figures 13C,D shows the results of the longitudinal analysis of sex and age interaction.

## 4. Summary and Concluding Discussion

In this paper we propose a novel framework for extracting surface-based local EA-CSF measurements from MR brain images. The proposed framework is the first to address the problem of obtaining local EA-CSF measurements in a way that is suitable for localized surface-based analysis. The proposed processing relies on a probabilistic tissue segmentation approach to generate a CSF probability map that is used to reconstruct the outer CSF hull surface. A Laplacian partial differential equation is solved between the inner cortical surface and the CSF hull surfaces to generate a vector field that is used to create streamlines connecting the surfaces at sub-voxel accuracy via a fourth-order Runge-Kutta approach. The starting and ending segments of the streamlines are then processed to fit them correctly within the boundary of the defined inner and outer surfaces. Along these streamlines, the CSF probability values are accumulated to quantify EA-CSF measures at each vertex on the cortical surface mesh. The proposed local EA-CSF extraction tool was used to study early postnatal brain development in typically developing infants from the IBIS dataset. Due to a high within-subject correlation, a longitudinal linear mixed model is proposed to incorporate fixed effects of age and sex as well as sex and age interactions.

The experimental results obtained from the scan/rescan human dataset show that the proposed local EA-CSF measure is reliable in a scan/rescan setting and produces reasonably stable results. The stability of the proposed method is further confirmed by the consistency in local EA-CSF patterns across the 3-time points used in studying local EA-CSF trajectories in the first 2 years of infancy. The experimental results reveals several findings through the proposed processing and analysis pipeline. First, local EA-CSF in several cortical regions, mainly in the frontal lobe areas, shows a statistically significant negative correlation with age. The longitudinal analysis also reveals several cortical regions with statistically significant higher local EA-CSF in males compared to females. Most of these regions also show a more substantial decrease in local EA-CSF across age. However, few cortical areas show higher negative local EA-CSF change rate in male subjects compared to females. Such localized findings confirm that the proposed local EA-CSF extraction pipeline reveals specific regions of significant change that would not be possible to be observed using the previous global EA-CSF approach.

The quantification of local EA-CSF measurements relies on streamlines that are generated based on solving an isotropic Laplace PDE between inner and outer surfaces on a created voxel grid. However, such isotropic PDE solution is purely based on the boundaries implied by the inner and outer surfaces. Hence, a limitation of the proposed framework is that the generated streamlines are not constrained to areas containing CSF. The method also fails to account for partial volume effects that lie between the two boundaries. In the future, we plan to improve the proposed local EA-CSF quantification by replacing the isotropic Laplace PDE with an anisotropic version (Joshi et al., 2018) in which the diffusion coefficient varies spatially in proportion to the fraction of CSF in each voxel. This allows for generating streamlines that follow a realistic path through areas containing CSF, leading to improvements in the accuracy and reliability of extracted local EA-CSF measurements.

## Data Availability Statement

The source code and scripts of our method will be released as open source with the next version of the Auto-EACSF tool on github (ongoing development). All tools developed at the UNC Neuro Image Research and Analysis Laboratory are open source. All raw MRI datasets and associated demographic information employed in this study is available at: NIH/NDA: https://nda.nih.gov/edit_collection.html?id=19.

## Ethics Statement

The studies involving human participants were reviewed and approved by the Institutional Review Boards (IRB) of all participating institutions. Written informed consent to participate in this study was provided by the participants' legal guardian/next of kin.

## Author Contributions

Method development and computational analysis was mainly performed by MM under guidance by MAS and MDS, with additional guidance and methodological input by GG and SP. Parts of processing was performed by SK and JG. Study design, data acquisition was performed by AE, SD, AE. RM, KB, RS, HH, and JP. Interpretation of results was performed by MM, MAS, and MDS. Document preparation was performed by all co-authors.

## Conflict of Interest

The authors declare that the research was conducted in the absence of any commercial or financial relationships that could be construed as a potential conflict of interest.
